# Siblings versus parents and friends: longitudinal linkages to adolescent externalizing problems

**DOI:** 10.1111/jcpp.12049

**Published:** 2013-02-12

**Authors:** Ivy N Defoe, Loes Keijsers, Skyler T Hawk, Susan Branje, Judith Semon Dubas, Kirsten Buist, Tom Frijns, Marcel AG van Aken, Hans M Koot, Pol AC van Lier, Wim Meeus

**Affiliations:** 1 Department of Developmental Psychology, Utrecht UniversityUtrecht; 2 Research Centre for adolescent Development, Utrecht UniversityUtrecht; 3 Research Centre Psychosocial Development in Context, Utrecht UniversityUtrecht; 4 Trimbos-InstituteUtrecht; 5 Department of Developmental Psychology, VU University Amsterdam; 6 Developmental Psychology, Tilburg UniversityTilburg, The Netherlands

**Keywords:** Externalizing problems, siblings, longitudinal, negative interaction, adolescents, friends, parents

## Abstract

**Background:** It is well documented that friends’ externalizing problems and negative parent–child interactions predict externalizing problems in adolescence, but relatively little is known about the role of siblings. This four-wave, multi-informant study investigated linkages of siblings’ externalizing problems and sibling–adolescent negative interactions on adolescents’ externalizing problems, while examining and controlling for similar linkages with friends and parents.

**Methods:** Questionnaire data on externalizing problems and negative interactions were annually collected from 497 Dutch adolescents (*M* = 13.03 years, *SD* = 0.52, at baseline), as well as their siblings, mothers, fathers, and friends.

**Results:** Cross-lagged panel analyses revealed modest unique longitudinal paths from sibling externalizing problems to adolescent externalizing problems, for male and female adolescents, and for same-sex and mixed-sex sibling dyads, but only from older to younger siblings. Moreover, these paths were above and beyond significant paths from mother–adolescent negative interaction and friend externalizing problems to adolescent externalizing problems, 1 year later. No cross-lagged paths existed between sibling–adolescent negative interaction and adolescent externalizing problems.

**Conclusions:** Taken together, it appears that especially older sibling externalizing problems may be a unique social risk factor for adolescent externalizing problems, equal in strength to significant parents’ and friends’ risk factors.

Youths’ externalizing problems, such as delinquency and aggression, negatively and directly affect the external environment and can result in great economic costs and social distress. A notable increase in delinquency is evident in adolescence (Farrington, 1986) primarily entailing minor delinquent acts (i.e., vandalism and shoplifting) (Moffitt, 1993). Several variants of social learning theory (Bandura, 1977) suggest that adolescents’ externalizing problems occur via modeling of such behaviors from their social environment (e.g., deviancy training; Dishion, Spracklen, Andrews & Patterson, 1996). In addition, coercion theory posits that the *quality* of adolescent relationships with significant others may increase adolescents’ likelihood of externalizing problems (Patterson, 1982). Both of these theories are prominent ‘social learning’ explanations for friend and parent influences on adolescent externalizing problems. Remarkably, although most families consist of multiple children (e.g., Statistics Netherlands, 2003), evidence for sibling influences on adolescent externalizing problems is relatively scarce (Dunn, 2005), even though they are theoretically just as likely as friend and parent influences. Furthermore, the sibling–adolescent, friend–adolescent, and parent–adolescent subsystems are interrelated (Bronfenbrenner, 1979), implying that sibling associations should also be considered when studying friends’ or parents’ associations with adolescents’ externalizing problems. The current multi-informant, 4-year study draws upon social learning theories of friend and parental influences to examine the unique and relative roles of siblings’ externalizing problems and/or negative interactions with target youths in predicting externalizing problems in adolescence.

## Sibling externalizing problems versus friend and parent externalizing problems

Social learning theory (Bandura, 1977) postulates that people learn each other’s behaviors through observation and imitation. Variants of such modeling theories are usually applied to the friend context, to explain the development and maintenance of similarity in externalizing problems among friends. For example, *deviancy training* theory posits that mutual social processes (e.g., laughing at antisocial acts) among individuals engaged in externalizing problem behaviors may reinforce adolescents’ deviant behaviors, and proposes that such behaviors can be learned (Dishion et al., 1996). Likewise, similarity between parents’ and adolescents’ externalizing problems could be the result of youths learning these behaviors from parents (e.g., Thornberry, Freeman-Gallant, Lizotte, Krohn & Smith, 2003). Considering the aforementioned accounts of behavioral modeling in friend–adolescent and parent–adolescent relationships, it is likely that similar processes also occur between adolescents and siblings who exhibit externalizing problems.

Numerous cross-sectional studies demonstrate significant associations between siblings’ externalizing problems (e.g., Craine, Tanaka, Nishina & Conger, 2009; Lauritsen, 1993). One of the few longitudinal studies among early adolescents showed that older siblings’ externalizing problems were related to a faster increase in younger siblings’ externalizing problems 2 years later within same-sex sibling dyads (Buist, 2010). Similarly, another study showed that older siblings’ antisocial behavior at ages 10 and 12 predicted younger siblings’ antisocial behavior at age 16 (Compton, Snyder, Schrepferman, Bank & Shortt, 2003). Hence, these findings suggest that modeling and imitation of externalizing problem behaviors may also occur between siblings.

## Negative interactions with sibling versus negative interactions with parents and friends

Close relationships characterized by negative interactions can also be a predictor of adolescent externalizing problems. Patterson’s (1982) *coercion theory* posits that coercive processes between parents and children provide a training ground for adolescents’ externalizing problem development. In support of coercion theory, one recent longitudinal study reported a bidirectional relationship between parent–adolescent negative interaction and youths’ externalizing problems (Burt, McGue, Krueger & Iacono, 2005). Moreover, these processes may also be evident in the friend context, at least in childhood. For example, one study documented that coercive friend interactions were related to more externalizing problems (Snyder et al., 2008).

Similar processes may also occur in the sibling dyad. Patterson’s coercion theory postulates that ‘sibling training in coercion’ can emerge when parent–child negative interactions spill over to the sibling dyad, resulting in siblings’ externalizing problems (Patterson, 1984, 1986). Empirical research on sibling negative interactions shows that they are typical and frequent, but decline after early adolescence (Kim, McHale, Wayne Osgood & Crouter, 2006) and may predict adolescent externalizing problems. For instance, a study demonstrated that sibling negative interactions at ages 10–12 predicted adolescent externalizing problems over 4 years (Bank, Burraston & Snyder, 2004; see also, Criss & Shaw, 2005; Natsuaki, Ge, Reiss & Neiderhiser, 2009; Slomkowski, Rende, Conger, Simons & Conger, 2001).

Collectively, empirical literature shows that the social learning perspective (Bandura, 1977) and coercion theory (Patterson, 1984) describe meaningful social processes with parents and friends who contribute to adolescent externalizing problems. In contrast, sibling relationships have been relatively understudied. The present research aims to extend the current literature by establishing whether these theories are also relevant for sibling relationships. We also address several of the methodological limitations in previous research.

## Addressing methodological issues

First, the sibling dyad should not be studied in isolation, because that would ignore previously established interrelations between the sibling, parent, and friend subsystems (Bronfenbrenner, 1979; for a review: Steinberg & Morris, 2001). For instance, although parental differential treatment is possible, parents’ behaviors might still have identical consequences for all siblings as a group as they share the same parents (e.g., Boyle et al., 2004). Accordingly, sharing the same antisocial parents might cause siblings to overlap in externalizing problems; this may reflect both genetic and social learning processes (Boyle et al., 2004; Thornberry et al., 2003). Siblings’ similarity in externalizing problems may also stem from their communal antisocial friends (Rowe & Gulley, 1992). Thus, it is necessary to disentangle unique sibling associations from those of friends and parents.

Second, some studies have accounted for parent–child negative interactions (i.e., Bank et al., 2004; Criss & Shaw, 2005; Natsuaki et al., 2009). However, none to our knowledge have longitudinally examined whether sibling negative interactions contribute to adolescent externalizing problems, independent of the *simultaneous* influences of both parent–adolescent and friend–adolescent negative interactions.

Third, the gender and birth-order composition of sibling dyads may moderate sibling associations. It is a commonly held notion that same-sex pairs exert stronger influence on each other, although mixed-sex sibling pairs may also overlap in their externalizing problems (e.g., Buist, 2010). Furthermore, although social learning theory (Bandura, 1977) suggests reciprocal relationships between persons involved, older siblings may have stronger effects, because they are more likely already involved in delinquency (Farrington, 1986) and may thus serve as role models for antisocial behavior (Moffitt, 1993). Indeed, the majority of research on birth order in sibling dyads reports a unidirectional relationship, from older siblings’ externalizing problems to younger siblings’ externalizing problems (e.g., Buist, 2010; Craine et al., 2009).

Fourth, in general, past research has rarely controlled for transactional processes (Granic & Patterson, 2006) in which parents, friends, and siblings not only affect adolescents but are also affected *by* adolescents. For instance, reciprocal links have been established between friends’ and adolescents’ externalizing problems (e.g., Haynie & Osgood, 2005), and between poorer quality parent–child relationships and adolescent externalizing problems (e.g., Lytton, 1990; Keijsers, Loeber, Branje & Meeus, 2011). Thus, to disentangle whether siblings predict externalizing problems, it is necessary to control for reverse associations.

### The present study

In sum, the primary goal of this multi-informant, 4-year study was to establish the roles of siblings’ externalizing problems and negative interaction in the prediction of adolescent externalizing problems, while estimating and controlling for similar links with parents and friends. In addition, reverse links from adolescents’ externalizing problems to the relationships with and externalizing problems of siblings, friends, and parents were also assessed and controlled for. Based on our dual theoretical approach, we hypothesized that both sibling externalizing problems and negative interactions would uniquely predict adolescent externalizing problems, beyond the hypothesized similar linkages from parents and friends. Finally, we explored moderation by adolescent’s gender, and by gender and birth-order composition in the sibling dyad.

## Method

### Participants

Participants were recruited from the project ‘Research on Adolescents Development And Relationships’ (RADAR; see for instance: Keijsers et al., 2012), a prospective longitudinal study in the Netherlands. Four annual waves of questionnaire data were analyzed from 497 targeted Dutch adolescents (57% male and 43% female), along with their siblings, fathers, mothers, and self-nominated best friends. These youths predominantly (89%) came from families with a medium or high socioeconomic status (SES), with a remaining 11% from families with low SES (Statistics-Netherlands, 1993).

At baseline (T1), target adolescents were 13.03 years (*SD* = .52), siblings were 14.92 years (*SD* = 3.33), fathers were 46.76 years (*SD* = 5.12), and mothers were 44.46 (*SD* = 4.50) years, on average. A total of 408 sibling dyads were present: 111 brother–brother pairs, 100 sister–sister pairs, 122 brother–sister pairs, and 75 sister–brother pairs. In addition, 288 target adolescents were younger than their siblings, and 115 adolescents were older.

Approximately 92% of target adolescents had a participating best friend (*M*_Age_ = 13.17, *SD* = .84) at T1, and 79% had a best friend participating each year. Friendships were quite stable: From T1 to T2, 69% of the adolescents nominated the same person as their best friend (79% from T2 to T3 and 66% from T3 to T4).

### Procedure

Families received a description of the RADAR project and a written informed consent document. In addition, the target adolescent was asked to invite and to provide contact information of his/her best friend. Once informed consent was granted by target adolescents, best friends, and parents of these adolescents, a trained research assistant arranged home visits to administer the questionnaires to the respondents. Families received a total equivalent to US $100 per home visit. Friends were paid US $35.

### Measures

*Externalizing problems* during the previous 6 months were assessed via self-reports. Adolescents, siblings, and friends reported on 30 items of the Youth Self Report (YSR: Achenbach, 1991). Mothers and fathers filled in the 35-item Adult Self Report (ASR: Achenbach & Rescorla, 2003). Both questionnaires contain items such as ‘I use drugs or alcohol’ and ‘I fight a lot’. Answers were given on 3-point Likert scales ranging from (0) *not true* to (2) *very true or often true*. Mean scores were calculated. For each wave, the externalizing problems scales of the YSR and ASR showed good reliability (see Table 1).

**Table 1:** Descriptive statistics, reliabilities, and 1-year stability (correlation coefficients)

	T1			T2			T3			T4			Relative Stability		
Variable	*M*	*SD*	α	*M*	*SD*	α	*M*	*SD*	α	*M*	*SD*	α	T1–T2	T2–T3	T3–T4
Externalizing problems															
Adolescent	.35	.24	.87	.32	.27	.91	.35	.32	.89	.35	.26	.89	.64**	.58**	.77**
Sibling	.37	.23	.87	.34	.20	.83	.31	.22	.85	.29	.21	.85	.66**	.73**	.70**
Mother	.12	.13	.83	.10	.11	.83	.09	.11	.84	.08	.09	.77	.72**	.76**	.73**
Father	.13	.12	.80	.13	.14	.86	.11	.13	.85	.10	.12	.83	.63**	.70**	.77**
Friend	.38	.22	.85	.36	.25	.88	.36	.27	.88	.35	.25	.88	.60**	.58**	.57**
Negative interaction															
Sibling	2.39	.79	.93	2.36	.81	.94	2.23	.81	.95	2.14	.82	.95	.63**	.66**	.72**
Mother	1.52	.53	.92	1.55	.54	.92	1.52	.50	.90	1.55	.56	.92	.69**	.69**	.71**
Father	1.51	.50	.90	1.52	.53	.92	1.51	.52	.91	1.53	.51	.92	.70**	.67**	.70**
Friend	1.25	.34	.80	1.26	.37	.83	1.27	.40	.85	1.28	.42	.85	.39**	.36**	.41**

Externalizing, externalizing problems.

** *p* < .01.

*Negative interactions* with the adolescent were reported by mothers, fathers, siblings, and friends, using the ‘Negative Interaction’ subscale of the Network of Relationships Inventory (NRI; Furman & Buhrmester, 1985). Negative interactions were assessed with a 5-point Likert scale ranging from 1 (*little to none*) to 5 (*could not be more*), and it comprises measures of conflict (three items; e.g., ‘How much do you and your sibling disagree and quarrel?) and antagonism (three items; e.g., ‘How much do you and your sibling hassle or nag one another?). Thus, higher scores indicate greater quantity (not intensity) of negative interactions. Mean scores across items were used. Reliabilities were acceptable across waves (Table 1).

### Strategy of analyses

To investigate the hypothesized longitudinal links from siblings’ externalizing problems and sibling–adolescent negative interactions to adolescent externalizing problems, we constructed a series of multi-informant cross-lagged panel models in M*plus* 6.1 (Muthén & Muthén, 1998). In step 1, we tested a model per significant other, resulting in four models. Each model contained four repeated measures of externalizing problems of the adolescent, externalizing problems of the significant other (i.e., sibling, friend, mother, or father), and of negative interactions between adolescents and that significant other. Hypothesized longitudinal links were examined from externalizing problems of – and negative interactions with – the significant other, at a given time point to adolescent externalizing problems 1 year later. We controlled for 1-year stability paths of each variable, all possible T1 associations, and all possible concurrent error covariance between variables at each measurement wave, and reverse longitudinal links (i.e., links from adolescent externalizing problems to externalizing problems of and negative interactions with the significant other, 1 year later).

Each model was time invariant, meaning that hypothesized cross-lagged paths and reverse cross-lagged paths could be constrained to be equal over time without worsening the model-fit (sibling: Wald χ^2^ (4) = 6.99, *p* = .14; mother: Wald χ^2^ (4) = 2.61, *p* = .63, father: Wald χ^2^ (4) = .51, *p* = .97; friend: Wald χ^2^ (4) = .15, *p* > .99). In addition, we examined whether members of the sibling–adolescent and friend–adolescent dyads were distinguishable (Kenny, Kashy & Cook, 2006), by testing whether the cross-lagged paths from siblings and friends to adolescents could be constrained to be equal to the reverse paths, from adolescents to siblings and friends. These constraints did not worsen the model fit (sibling: Wald χ^2^ (3) = 1.12, *p* = .77; friend: Wald χ^2^ (3) = 2.72, *p* = .44), and were thus added.

We tested for moderation of adolescent gender, by constraining parameters to be equal for males versus females in these preliminary models. In the sibling model, we additionally tested for moderation of gender composition (i.e., same-sex versus mixed-sex sibling dyads) and birth-order composition (i.e., older–younger sibling versus younger–older sibling dyads).

In step 2, we examined the relative strength of links of siblings, from parents and friends to adolescent’s externalizing problems. Therefore, we combined only the significant links in one final combined model.

Attrition in this study was low. Of the 497 families at T1, 466, 474, and 440 participated at the three follow-up measurements, respectively. Per variable, a maximum of 27% of the cases were missing. Missing data analyses suggested a random pattern of missingness. A Full Information Robust Maximum Likelihood Estimator was used for all models (Satorra & Bentler, 1994), because the data for our externalizing problems measure were somewhat skewed (Skewness was between 0.65 and 2.47). All models had a good fit to our data (see Online Supplementary Information concerning details about the fit of the preliminary models).

## Results

Descriptive statistics are provided in Table 1. Notably, the frequency of negative interaction was highest among siblings (compared with mothers, fathers, and friends). Moreover, mean levels for sibling–adolescent negative interactions decreased significantly over time (*F*(3,332) = 12.26, *p* < .01, η^2^ = .10). Bivariate concurrent correlations among the study variables within each measurement wave^1^ are shown in Tables 2 and 3.

**Table 2:** Bivariate concurrent correlations at times 1 and 2

	1	2	3	4	5	6	7	8	9
Externalizing problems									
Adolescent	–	.09	.21**	.05	.20**	.09	.33**	.22**	.16**
Sibling	.15**	–	.17**	.21**	.05	.30**	.11**	.17**	−.01
Mother	.24**	.07	–	.06	.07	.19**	.34**	.14**	.01
Father	.08	.20**	.07	–	.06	.04	.01	.28**	−.01
Friend	.23**	.06	.14**	−.01	–	−.01	.17**	.10*	.34**
Negative interaction									
Adolescent–sibling	.16**	.31**	.07	.09	.07	–	.21**	.20**	.08
Adolescent–mother	.34**	.05	.34**	−.03	.12**	.20**	–	.41**	.17**
Adolescent–father	.28**	.04	.15**	.16**	.08	.14**	.39**	–	.16**
Adolescent–friend	.22**	.02	−.00	.01	.24	.12*	.16**	.15**	–

The concurrent correlations of the first year are presented below the diagonal, and the concurrent correlations of the second year are displayed above the diagonal.

* *p* < .05

* *p* < .01.

**Table 3:** Bivariate concurrent correlations at times 3 and 4

		1	2	3	4	5	6	7	8	9
Externalizing problems										
Adolescent		–	.17**	.17**	.10*	.20**	.09	.28**	.27**	.15*
Sibling		.22**	–	.11**	.12*	.11*	.25**	.13**	.12*	.07
Mother		.24**	.18**	–	.04	.06	.08	.37*	.12*	.02
Father		.10*	.15**	−.00	–	.09	.00	.02	.16**	−.03
Friend		.23**	.06	.06	.03	–	−.02	.07	.05	.23**
Negative interaction										
Sibling		.14**	.18**	.07	.11*	−.02	–	.20**	.23**	.03
Mother		.24**	.04	.31**	.05	.06	.25**	–	.41**	−.01
Father		.23**	.11*	.08	.19**	.09	.31**	.43**	–	.10*
Friend		.16**	−.01	−.00	−.04	.19**	.09	.10*	.02*	–

The concurrent correlations of the third year are presented below the diagonal, and the concurrent correlations of the fourth year are displayed above the diagonal.

* *p* < .05

** *p* < .01.

### Preliminary models per significant other

In line with our hypothesis, siblings’ externalizing problems modestly predicted adolescent externalizing problems (βs between .04 and .05) in the sibling model, and the same was found for friends (βs between .04 and .05). In contrast, mothers’ and fathers’ externalizing problems did not predict adolescents’ externalizing problems in the parent models.

Contrary to our expectations, sibling negative interactions did not predict adolescent externalizing problems, nor were there reversed effects. However, adolescent negative interactions with mothers (βs between .06 and .07) and friends (βs between .04 and .05) significantly predicted adolescent externalizing problems. Father–adolescent negative interactions predicted adolescent externalizing problems at a trend level (βs between .04 and .05, *p* = .06). (see Online Supplementary Information for details concerning the preliminary analyses.)

### Multigroup comparisons

Multigroup analyses for adolescent gender revealed few gender differences. T1 associations in the sibling, mother, and father model were not moderated by gender (sibling: Wald χ^2^ (3) = 1.49, *p* = .68; mother: Wald χ^2^ (3) = .82, *p* = .85; father: Wald χ^2^ (3) = 1.51, *p* = .68), but T1 associations of friend externalizing problems with friend–adolescent negative interactions existed only in the model for males (males: β = .34, *p* < .01; females: β = .09, *p* = .21; Wald χ^2^ (3) = 12.78, *p* = .21). Cross-lagged paths of each model, per significant other, did not differ for males and females (sibling: Wald χ^2^ (4) = 8.48, *p* = .08; mother: Wald χ^2^ (4) = 2.19, *p* = .70; father: Wald χ^2^ (4) = 3.64, *p* = .46; friend: Wald χ^2^ (4) = 3.08, *p* = .54).

For the sibling model, we also examined whether gender and birth-order composition in the sibling dyad moderated the hypothesized paths. No differences for gender composition of the sibling dyad for T1 associations (Wald χ^2^ (3) = 2.98, *p* = .40), or cross-lagged paths (Wald χ^2^ (4) = 1.64, *p* = .80) were found. As for birth-order moderation, T1 associations were equal across groups (Wald χ^2^ (3) = 2.80, *p* = .42), but the cross-paths were moderated (Wald χ^2^ (4) = 10.50, *p* = .03). Siblings’ externalizing problems predicted adolescents’ externalizing problems only when siblings were older than adolescents (β’s between .04 and .05).

### The combined model: relative and unique sibling associations

**Figure 1:**
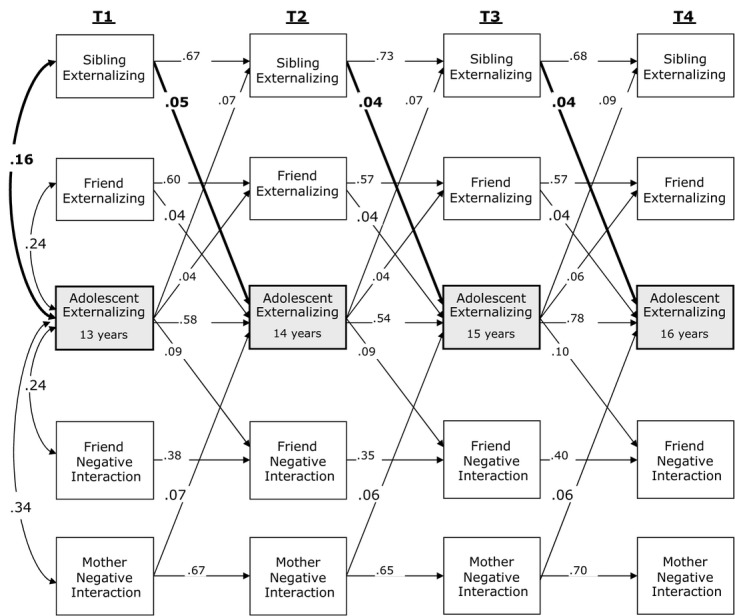
Significant standardized paths for combined model. Externalizing = externalizing problems. Bold arrows indicate hypothesized sibling paths. Concurrent error covariance at T2, T3, and T4 are estimated, but not depicted.

Finally, we investigated unique links of sibling externalizing problems compared with the significant links of friends, mothers, and fathers^2^ in one combined model (see Figure 1). Results of the combined model showed significant T1 associations (β = .16) between siblings’ externalizing problems and adolescents’ externalizing problems and significant cross-lagged effects of sibling externalizing problems on adolescent externalizing problems (β = .04–.05). Reverse associations from adolescent externalizing problems to sibling externalizing problems were also present (βs between .07 and .09).

Pertaining to friends and mothers, we found significant T1 associations of adolescent externalizing problems with friends’ externalizing problems (β = .24), friend–adolescent negative interactions (β = .24), and mother–adolescent negative interactions (β = .34). Friends’ externalizing problems (βs between .04 and .06) and mother–adolescent negative interactions (βs between .06 and .07) also remained significant predictors of adolescent externalizing problems in the combined model, but friend negative interaction was no longer a significant predictor. Reversed links were found from adolescent externalizing problems to friend externalizing problems (βs between .07 and .09), and from adolescent externalizing problems to friend negative interaction (βs between .09 and .10). Taken together, results showed that externalizing problem behaviors of siblings and friends and negative interaction with mothers were significant longitudinal predictors of adolescent externalizing problems.

## Discussion

Sibling relationships are one of the most constant social companionships, providing a proximal context for youth’s developmental opportunities (Jenkins & Dunn, 2009). Accordingly, siblings might influence adolescent behaviors in ways similar to both parents and friends. Moreover, prominent developmental theories (Bronfenbrenner, 1979) and previous empirical investigations (review: Steinberg & Morris, 2001) suggest that the sibling subsystem is interrelated with the parent–adolescent and friend–adolescent subsystems. Hence, sibling influences on adolescent externalizing problems may be partly explained by other social influences. To our knowledge, this study is the first to longitudinally examine the unique and relative role of siblings’ externalizing problems and sibling–adolescent negative interactions in the prediction of adolescent externalizing problems. Strict cross-lagged panel models revealed modest but unique cross-sectional and bidirectional longitudinal links between sibling externalizing problems and adolescent externalizing problems. Moreover, longitudinal links were similar in magnitude to links from friend externalizing problems and mothers–adolescent negative interactions to adolescent externalizing problems, both of which have more frequently been addressed in prior studies. Although T1 associations between sibling–adolescent negative interactions and adolescent’s externalizing problems were found, no longitudinal paths from sibling–adolescent negative interactions to adolescent externalizing problems were present. We address the implications of these findings below.

### Sibling externalizing problems

Modeling and imitation of behaviors are core components of social learning theory (Bandura, 1977). Considering that sibling relationships are among the most prominent social factors in adolescence (Jenkins & Dunn, 2009), we hypothesized that one sibling’s externalizing problems would predict those of the other sibling. Indeed, the primary finding of this study is that sibling externalizing problems (but not sibling negative interaction) could be a unique risk factor for subsequent adolescent externalizing problems, and vice versa, even when controlling for established roles of parents and friends. The current longitudinal study is the first to demonstrate robust links between siblings’ externalizing problems. That is, although a few studies have considered parent–adolescent or friend–adolescent associations when studying sibling similarity in externalizing problems (e.g., Fagan & Najman 2003; Natsuaki et al., 2009), we could find no other study accounting for *simultaneous* influences of parents *and* friends on adolescents. In addition, this study extends the relevance of social learning theory to the sibling dyad.

In line with a social learning perspective, we found reciprocal, positive linkages between siblings’ externalizing problems. This suggests that siblings may mimic each others’ externalizing problem behavior, fueling a downward spiral in which siblings mutually maintain and reinforce each other’s problematic behavior. This illustration corresponds to deviancy training among antisocial friends (Bandura, 1977; Dishion et al., 1996). However, unlike friends who often have the same age, siblings are almost always of a different age. Accordingly, findings revealed that older siblings’ externalizing problems predicted younger siblings’ externalizing problems but not the reverse. These results concur with the majority of sibling studies that report a typical direction of influence from older sibling to younger sibling (e.g., Buist, 2010; Craine et al., 2009). Hence, although deviancy training may also occur in sibling dyads, the direction of modeling is predominantly from the older to the younger sibling.

Interestingly, associations between siblings’ externalizing problems were comparable for male and female adolescents and for same-sex and mixed-sex sibling pairs. Thus, the present findings support the suggestion by Snyder, Bank and Burraston (2005) that modeling behaviors – while perhaps more likely for same-sex siblings (e.g., Buist, 2010) – can also occur within mixed-sex sibling pairs. These moderation results should be interpreted with caution, however, because we may not have had enough power to detect small moderation effects.

### Sibling negative interactions

Coercion theory posits that parent–child negative interactions may trigger negative interactions in the sibling dyad, which may predict externalizing problems in childhood and adolescence (Patterson, 1984, 1986). In the present research, sibling–adolescent negative interactions were associated, but not longitudinally. This contradicts previous empirical studies with at-risk samples, showing that sibling negative interactions predict adolescent externalizing problems (e.g., Bank et al., 2004; Criss & Shaw, 2005).

Several explanations can be given for this discrepancy. First, sibling negative interactions may be a normative process that declines after early adolescence (e.g., Kim et al., 2006; Kramer, 2010), and could be either destructive or constructive (Kramer, 2010). This study possibly tapped into the more constructive and normative patterns of negative interaction, including small disagreements. Second, our study used a very stringent methodological approach, including a longitudinal design controlling for reverse paths and temporal stability, and using multiple informants for different measures. Bivariate associations that did not take all of these possible confounds into account indeed showed the (small) positive correlations that we predicted. Future studies with a stringent longitudinal design are needed to test this hypothesis further.

## Limitations and implications

Despite the multi-informant longitudinal design of this study, there are also some limitations. First, the magnitudes of the cross-lagged paths were small. We believe this is caused by our rigorous cross-lagged panel design with different reporters for different variables. Effect sizes were comparable to a similar recent study (i.e., Natsuaki et al., 2009) on ‘Nonshared Environment in Adolescent Development’ data (Hetherington et al., 1999). This suggests the cross-lagged paths are small but meaningful (e.g., McCartney & Rosenthal, 2000). Second, although our sample size was adequate for this type of modeling, statistical power was perhaps limited for finding moderation effects. Third, our measurement for negative interaction did not make a distinction between constructive and destructive negative interaction. A conflict resolution measure might have better assessed whether constructive or destructive negative interaction was being tapped. Fourth, we relied purely on longitudinal questionnaire data, and did not directly study underlying mechanisms. Hence, no causal inferences can be made from the present, nonexperimental results. Finally, we postulated social learning as the mechanism underlying our findings, but other explanations for the sibling linkages may also be plausible, such as those derived from ‘identity based theories’ (see, e.g., Heilbron & Prinstein, 2008), as well as shared genes or gene-environment interactions.

## Conclusion

Despite the aforementioned limitations, this study overcame several methodological challenges unaddressed in prior research. It demonstrated the unique relation of older sibling’s externalizing problems with subsequent adolescent externalizing problems, independent of the interrelatedness between the sibling–adolescent, parent–adolescent, and friend–adolescent subsystems. Results suggest moreover that siblings and friends (i.e., peers) play a similar role in adolescent externalizing problems, as their problem behaviors are linked with adolescent externalizing problems to a similar extent. For parents, however, it was the relationship quality with adolescents – particularly mother–adolescent negative interaction – that predicted adolescent externalizing problems. Taken together, it appears that especially older sibling externalizing problems may be a unique social risk factor for adolescent externalizing problems, equal in strength to significant parents’ and friends’ risk factors.

**Key points**

- **What is known:** Parent–adolescent negative interaction and friend externalizing problems predict adolescent externalizing problems.
- However, relatively little is known about the sibling’s role in adolescent externalizing problems.
- **What is new:** Specifically older sibling externalizing problems predicted adolescent externalizing problems, above and beyond links from parent–adolescent negative interaction, friend–adolescent negative interaction, and friend externalizing problems to adolescent externalizing problems. The sibling dyad appears to provide a unique and relevant context for social learning, comparable in strength to the friend–adolescent and parent–adolescent dyads.
- **Clinical relevance:** Including siblings in adolescent therapy for externalizing problems should be considered more often.

## Notes

1 A complete correlation table is available from the first author on request.

2 Because the paths from father–adolescent negative interactions to adolescent externalizing problems were a trend at *p* = .06, we ran two different combined models, in one of which we also included this trend, and in one of which we left it out. Both models yielded similar results. That is, the magnitudes of the paths were equal. We decided to report the model without the trend effect.

## Supporting Information

Online appendix: Detailed results from the preliminary models (Word document).
