# Supplementary material for: Siblings versus parents and friends: longitudinal linkages to adolescent externalizing problems
Source: J Child Psychol Psychiatry. 2013 Feb 12;54(8):881–9. doi: 10.1111/jcpp.12049 (PMC3807608; doi:10.1111/jcpp.12049)
Supplement: Supplementary file 1 — Online appendix: Detailed results from the preliminary models (Word document). [file jcpp0054-0881-sd1.doc]

Online Supporting Information for Defoe et al. (2013), Siblings versus parents and friends: longitudinal linkages to adolescent externalizing problems, doi:10.1111/jcpp.12049

**Detailed Results from the Preliminary Models**

**Contributions of Externalizing Problems of Siblings, Friends, Mothers and Fathers**

The preliminary cross-lagged panel models were specified separately for sibling, friend, mother and father (see Table 1). With respect to the sibling model presented in the first three columns, hypothesized linkages were found between adolescents’ externalizing problems of siblings and adolescents. That is, significant T1 associations (β = .16) were present between sibling externalizing problems and adolescent externalizing problems. In contrast, we found no significant correlated change between adolescent and sibling externalizing problems. In addition, we found modest yet significant positive cross-lagged paths from sibling externalizing problems to adolescents externalizing problems, indicating that higher relative levels of siblings’ externalizing problems predicted adolescent externalizing problems one year later (βs between .04 and .05). Modest reversed effects from adolescent externalizing problems to sibling externalizing problems were also present (βs between .07 and .09).

We found similar results for the friend model. Friends’ externalizing problems and adolescent externalizing problems were significantly correlated (β = .24). Significant correlated change was present at T2 (β = .11), and T3 (β = .16), but not at T4. Significant positive cross-lagged effects were also found from friend externalizing problems to adolescent externalizing problems (βs between .04 and .05) and vice-versa (βs between .05 and .06).

Results for the parent models showed positive T1 associations for the mother model (β = .24) but not for the fathers model. Correlated change was not found for mothers or fathers. Further, no hypothesized cross-lagged effects were present from parents’ externalizing problems to adolescent externalizing problems. Positive reversed effects were found in both the mother (βs between .05 and .09) and father models (βs between .04 and .06), however, indicating that adolescent externalizing problems positively predicted their parents’ externalizing problems.

Taken together, and in line with our sibling hypothesis, siblings’ externalizing problems modestly predicted adolescent externalizing problems, when also accounting for adolescents’ negative interactions with siblings. Additionally, friends’ externalizing problems as opposed to mothers’ and fathers’ externalizing problems predicted adolescents’ externalizing problems.

**Contributions of Adolescent Negative Interaction with Siblings, Friends, Mothers and Fathers**

Results are shown in Table 1. With regard to the sibling model, there was a significant T1 association between sibling negative interaction and adolescent externalizing problems (*β* = .18), but no correlated change was present. In contrast to our expectations, no significant cross-lagged effects existed from sibling negative interaction to adolescent externalizing problems, nor were there reversed effects.

Results of the friend model showed positive T1 associations (β = .23) between friend-adolescent negative interaction and adolescent externalizing problems, but no significant correlated change. Significant and positive bidirectional cross-lagged linkages were present from friend-adolescent negative interaction to adolescent externalizing problems, with βs between .04 and .05 for the path from friend-adolescent negative interaction to adolescents’ externalizing problems, and βs between 0.09 and .10 for the reversed paths.

For the mother and father models, positive T1 associations (β = .34 and β = .29 respectively) were present between parent-adolescent negative interaction and adolescent externalizing problems, as well as correlated change (βs ranging from .16 to .20 and βs from .13 from to .22 respectively). In both the mother and father models, positive cross-paths were found (βs between .06 and .07; βs between .04 and .05 respectively) from parent-adolescent negative interaction to adolescent externalizing problems (for fathers at a trend level *p* = .06). Significant reversed effects from parent negative interaction were present in the father model (βs between .05 and .06), but not in the mother model.

Thus contrary to our expectations concerning sibling effects, sibling negative interaction did not predict adolescent externalizing problems in the sibling model when simultaneously controlling for sibling externalizing problems. In addition, adolescent negative interactions with mother and friend (but not with father) significantly predicted adolescent externalizing problems.

Table 1

Preliminary Models: Longitudinal Cross-lagged Associations of Adolescent’s Externalizing Problems, with Negative Interactions and Externalizing Problems of Significant Others

|  | Sibling Model | | |  | Friend Model | | |  | Mother Model | | |  | | Father Model | | | | |
| --- | --- | --- | --- | --- | --- | --- | --- | --- | --- | --- | --- | --- | --- | --- | --- | --- | --- | --- |
| Model Parameters | *B* | *SE* | Β |  | *B* | *SE* | Β |  | *B* | *SE* | Β | |  | *B* | | *SE* | | Β |
| T1 Associations & Correlated Change of Externalizing Problems |  |  |  |  |  |  |  |  |  |  |  | |  |  | |  | |  |
| T1 Externalizing Problems – T1 Adolescent Externalizing Problems | .01 | .00 | .16** |  | .01 | .00 | .24** |  | .01 | .00 | .24** | |  | .00 | | .00 | | .07 |
| T2 Externalizing Problems – T2 Adolescent Externalizing Problems | -.00 | .00 | -.04 |  | .00 | .00 | .11* |  | .00 | .00 | .03 | |  | .00 | | .00 | | -.02 |
| T3 Externalizing Problems – T3 Adolescent Externalizing Problems | .00 | .00 | .06 |  | .01 | .00 | .16* |  | .00 | .00 | .11 | |  | .00 | | .00 | | .07 |
| T4 Externalizing Problems– T4 Adolescent Externalizing Problems | .00 | .00 | .04 |  | .00 | .00 | .02 |  | .00 | .00 | -.01 | |  | .00 | | .00 | | .01 |
| Hypothesized Cross-Lagged Paths of Externalizing Problems: |  |  |  |  |  |  |  |  |  |  |  | |  |  | |  | |  |
| T1 Externalizing Problems 🡪 T2 Adolescent Externalizing Problems | .06 | .02 | .05** |  | .05 | .02 | .04** |  | .02 | .06 | .01 | |  | .03 | | .04 | | .01 |
| T2 Externalizing Problems 🡪 T3 Adolescent Externalizing Problems | .06 | .02 | .04** |  | .05 | .02 | .04** |  | .02 | .06 | .01 | |  | .03 | | .04 | | .01 |
| T3 Externalizing Problems 🡪 T4 Adolescent Externalizing Problems | .06 | .02 | .05** |  | .05 | .02 | .05** |  | .02 | .06 | .01 | |  | .03 | | .04 | | .01 |
| Externalizing Problems  Reversed Cross-Lagged Paths |  |  |  |  |  |  |  |  |  |  |  | |  |  | |  | |  |
| T1 Adolescent Externalizing Problems🡪 T2 Externalizing Problems | .06 | .02 | .07** |  | .05 | .02 | .05** |  | .03 | .01 | .05** | |  | .02 | | .01 | | .04* |
| T2 Adolescent Externalizing Problems 🡪 T3 Externalizing Problems | .06 | .02 | .08** |  | .05 | .02 | .05** |  | .03 | .01 | .06** | |  | .02 | | .01 | | .04* |
| T3 Adolescent Externalizing Problems 🡪 T4 Externalizing Problems | .06 | .02 | .09** |  | .05 | .02 | .06** |  | .03 | .01 | .09** | |  | .02 | | .01 | | .06* |
| T1 Associations & Correlated Change of Negative Interaction |  |  |  |  |  |  |  |  |  |  |  | |  |  | |  | |  |
| T1 Negative Interaction – T1 Adolescent Externalizing Problems | .04 | .01 | .18** |  | .02 | .01 | .23** |  | .04 | .01 | .34** | |  | .03 | | .01 | | .29** |
| T2 Negative Interaction – T2 Adolescent Externalizing Problems | .01 | .01 | .06 |  | .00 | .00 | .05 |  | .01 | .00 | .16** | |  | .01 | | .00 | | .13* |
| T3 Negative Interaction – T3 Adolescent Externalizing Problems | .01 | .01 | .04 |  | .01 | .01 | .06 |  | .02 | .01 | .20** | |  | .02 | | .01 | | .22** |
| T4 Negative Interaction – T4 Adolescent Externalizing Problems | .01 | .01 | .06 |  | .00 | .00 | .02 |  | .01 | .00 | .16** | |  | .01 | | .00 | | .18* |
| Hypothesized Cross-Lagged Paths of Negative Interaction |  |  |  |  |  |  |  |  |  |  |  | |  |  | |  | |  |
| T1 Negative Interaction 🡪 T2 Adolescent Externalizing Problems | -.01 | .01 | -.02 |  | .04 | .02 | .05* |  | .04 | .02 | .07** | |  | .02 | | .01 | | .05 |
| T2 Negative Interaction 🡪 T3 Adolescent Externalizing Problems | -.01 | .01 | -.02 |  | .04 | .02 | .04* |  | .04 | .02 | .06** | |  | .02 | | .01 | | .04 |
| T3 Negative Interaction 🡪 T4 Adolescent Externalizing Problems | -.01 | .01 | -.02 |  | .04 | .02 | .05* |  | .04 | .02 | .06** |  | | .02 | .01 | | .04 | |
| Reversed Cross-Lagged Paths of Negative Interaction |  |  |  |  |  |  |  |  |  |  |  | |  |  | |  | |  |
| T1 Adolescent Externalizing Problems 🡪T2 Negative Interaction | .12 | .07 | .04 |  | .13 | .05 | .09** |  | .08 | .04 | .03 | |  | .10 | | .04 | | .05* |
| T2 Adolescent Externalizing Problems🡪T3 Negative Interaction | .12 | .07 | .04 |  | .13 | .05 | .09** |  | .08 | .04 | .04 | |  | .10 | | .04 | | .05* |
| T3 Adolescent Externalizing Problems🡪 T4 Negative Interaction | .12 | .07 | .05 |  | .13 | .05 | .10** |  | .08 | .04 | .04 | |  | .10 | | .04 | | .06* |

*Note*. One year stability paths were also estimated in the models, but they are not depicted in the table. Two-year stability for mothers’ and fathers’ Externalizing Problems were included as well (T1-T3; β = .20 & .41 T2-T4; β = .38 and.25 respectively), in order to increase model fit of the father model and to ensure similarity between the mother and father models. Model fit sibling model: χ^2^ (*df ) =* 145.05* (42)*,*  χ^2^ / *df =* 3.45, TLI = .91 ; CFI= .94; RMSEA = .06; Model fit friend model: 111.46** (42), χ^2^ / *df =* 2.65, TLI = .91, CFI= .94, RMSEA = .06; Model fit mother model: 141.54** (39), χ^2^ / *df =* 3.63, TLI = .92, CFI= .95, RMSEA = .07; Model fit father model: 119.87** (39), χ^2^ / *df =* 3.07, TLI = .92, CFI= .95, RMSEA = .07.

*p < .05; **p<.01.
